# Influence of Waste Rubber Powder on the Mechanical and Abrasion Resistance Properties of Concrete

**DOI:** 10.3390/ma18225157

**Published:** 2025-11-13

**Authors:** Shuangxi Li, Dongzheng Yu, Chunmeng Jiang, Zhimin Feng, Mai Zhao, Zhong Li

**Affiliations:** 1 College of Hydraulic and Civil Engineering, Xinjiang Agricultural University, Urumqi 830052, China; 2 Xinjiang Tianze Engineering Management Co., Ltd., Shihezi 832003, China

**Keywords:** rubber powder, hydraulic concrete, impact resistance, abrasion resistance

## Abstract

In the river environments of Xinjiang characterized by high sediment content and high flow velocities, hydraulic concrete is highly susceptible to damage from the impact and abrasion of bed load. Consequently, this imposes more stringent requirements on its mechanical properties and abrasion resistance. The incorporation of crumb rubber, a recyclable material, into concrete presents a dual benefit: it enables resource recycling while simultaneously offering a novel pathway for the development of concrete technology. This study takes rubber powder concrete as the research object. With the same water-to-binder ratio, rubber powder was incorporated at three volume fractions: 0%, 5%, and 10% of the cementitious material. The drop weight impact test and underwater steel ball method are adopted to evaluate its impact resistance and anti-scouring-abrasion performance, respectively. By testing the compressive strength, impact toughness, wear rate, anti-scouring-abrasion strength and three-dimensional morphological characteristics, the influence of rubber powder content on the mechanical properties and anti-scouring-abrasion performance of concrete is systematically analyzed. The research results show that the addition of rubber powder reduces the compressive strength of concrete, but significantly improves its impact resistance and anti-scouring-abrasion performance. Among all test groups, the concrete with 10% rubber powder content has the most significant decrease in compressive strength, with a decrease of about 37% compared with the 5% content group, while the 5% content group has a decrease of about 27% compared with the control group. However, its impact toughness at 3d, 7d and 15d is increased by about 84.7%, 88.4% and 84.4%, respectively, compared with the control group, showing the largest improvement range. At the same time, the wear rate of this group is reduced by about 42.5%, and the anti-scouring-abrasion strength is increased by about 61%. Combined with the three-dimensional morphology analysis, it can be seen that the specimens in this group exhibit the optimal anti-scouring-abrasion performance. In terms of microstructure, the porosity of rubber powder concrete increases, the generation of C-S-H gel decreases and its continuity is damaged, leading to a significant decrease in compressive strength. The reduction in the generation of delayed ettringite enhances the toughness and anti-scouring-abrasion performance. In general, the increase in rubber powder content will lead to a decrease in the compressive strength of concrete, but within a certain range, it can significantly improve its impact resistance and anti-scouring-abrasion performance. Crumb rubber effectively enhances the impact and abrasion resistance of hydraulic concrete, demonstrating strong application potential in high-flow, sediment-laden river environments.

## 1. Introduction

In river regions with high sediment content and high flow velocity (such as the Yellow River Basin and sand-laden river areas in Northwest China), the bed load and suspended load carried by the water flow exert intense impact and scouring-abrasion damage on hydraulic concrete structures. This type of damage is often coupled with the inherent brittleness and cracking issues of concrete itself, significantly exacerbating the functional degradation of the flow-passing surfaces of water-releasing structures and the risk of structural failure [1,2]. The continuous development of coupled damage not only substantially increases the cost of later maintenance and reinforcement, but also poses a severe threat to the long-term safe operation of water conservancy projects. In extreme cases, it may even lead to catastrophic accidents such as structural collapse. Therefore, in response to the long-service-life demands of water conservancy infrastructure in sand-laden river environments, the development of hydraulic concrete with high anti-scouring-abrasion and high impact resistance has become a crucial engineering and technical issue that urgently needs to be addressed at present [3,4].

Currently, commonly used anti-abrasion techniques for hydraulic concrete include the application of high-strength concrete, the addition of abrasion-resistant aggregates (such as steel fibers, polypropylene fibers, etc.), and surface coating treatments. However, although these methods improve the abrasion resistance of concrete to some extent, they still face issues such as high cost, complex construction, and insufficient durability. Therefore, developing a new type of abrasion-resistant concrete material that is both economical and efficient holds significant engineering importance.

As a typical industrial solid waste, the annual output of waste tires continues to rise with the rapid development of the rubber industry and automotive sector. According to industry statistics, the global annual generation of waste tires currently ranges from 25 to 30 million tons [5,6]. The accumulation and landfilling of large quantities of waste tires not only occupy land resources but also pose potential environmental risks such as soil and groundwater contamination. However, from the perspective of resource recycling, these readily available and low-cost waste rubber materials provide an abundant raw material source for the development and application of rubberized concrete, creating favorable conditions for synergistically advancing solid waste utilization and construction material innovation [7,8].

Rubberized concrete is a cement-based composite material prepared by partially replacing conventional aggregates with waste rubber particles (or rubber powder). It offers multiple advantages in terms of technical performance and application potential:

(1) Although the incorporation of rubber powder does not alter the chemical properties of cement hydration products, it optimizes the internal packing structure of concrete, improves workability, significantly reduces brittleness, and enhances toughness, impact resistance, and crack resistance. This strengthens the material’s energy absorption capacity under dynamic loads (e.g., earthquakes, explosions). Additionally, rubberized concrete exhibits functional properties such as lightweight, sound insulation, and thermal insulation, enabling potential applications in seismic-resistant structures and road noise reduction [9,10,11,12].
(2) This technology enables high-value resource utilization of waste rubber, offering both significant environmental benefits (reducing solid waste pollution) and economic advantages (lowering construction material costs).
(3) Processing waste tires into rubber powder for use in concrete can effectively improve the abrasion resistance of the material, providing a new technical approach to mitigating abrasion damage in hydraulic concrete subjected to medium- to high-speed sediment-laden flow [13,14].

Existing research has confirmed the material’s performance potential: Zhai et al. [15] experimentally demonstrated that as the rubber particle content increases, the abrasion resistance of concrete first increases and then decreases, with an optimal content leading to an improvement of over 14.9% compared to conventional concrete. S.S. Rajahrm et al. [16] investigated the influence of water-to-cement ratio and aggregate type on the abrasion resistance of concrete. Using the wear rate at a 3.5 mm abrasion depth as an indicator, they revealed the correlation between abrasion resistance strength and water-to-cement ratio, identifying a peak performance within a specific range, though the criteria for determining the optimal value require further clarification. Other researchers, employing drop-weight impact tests, found that increasing rubber content can enhance the fracture toughness of concrete by more than 30%. These results collectively indicate that rubberized concrete holds significant engineering application prospects in hydraulic structures and other related fields [17,18,19,20,21].

However, crumb rubber concrete is not without its drawbacks. Compared to conventional concrete, its compressive and tensile strengths are significantly reduced. N.A. Siddique et al. investigated the mechanical properties of waste rubber-modified concrete and found that when the rubber content reached 50%, the compressive and tensile strengths decreased by up to 70% [22]. This degradation in mechanical strength remains a major factor limiting the widespread application of crumb rubber concrete.

Under this research background, this study investigates rubberized concrete with crumb rubber contents (0%, 5%, 10%), using plain concrete as the control. Macroscopically, abrasion resistance was evaluated through underwater steel ball tests (measuring wear rate and abrasion strength) combined with 3D topography analysis. Impact resistance was assessed via drop-weight tests determining impact toughness. Microscopically, scanning electron microscopy (SEM) characterized pore structures and hydration products to reveal microstructural effects.

## 2. Materials and Methods

### 2.1. Materials

**Figure 1:**
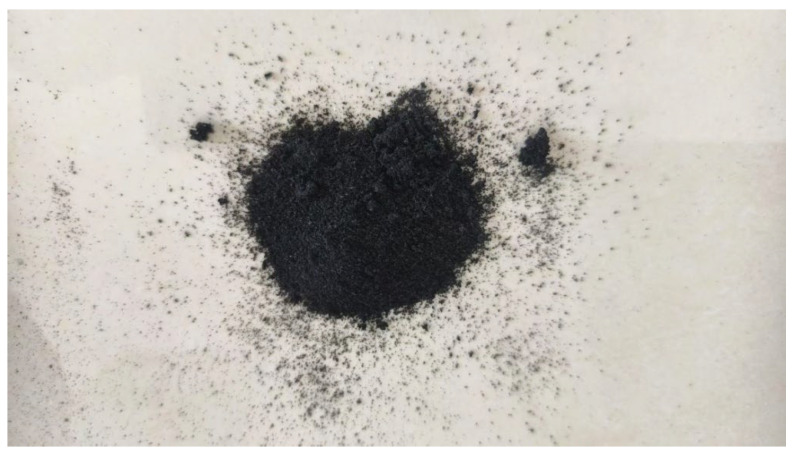
Microscopic image of the waste rubber powder (40 mesh) used in the study.

(1) Cement: Ordinary Portland cement (P·O 42.5R) produced by Xinjiang Tianshan Cement Plant (Urumqi, China), with physical property indicators shown in Table 1.

(2) Fine Aggregate: Natural medium sand with a fineness modulus of 2.9 was used as the fine aggregate, and its physical properties are presented in Table 2.

(3) Coarse Aggregate: Continuously graded limestone crushed stone with a particle size range of 5–20 mm was used as the coarse aggregate, and its physical property indices are presented in Table 3.

(4) Rubber Powder: Waste rubber powder (particle size: 40 mesh, 0.425 mm) sourced from Dujiangyan Huayi Rubber Co., Ltd. (Wuhan, China) was used, as shown in Figure 1. Table 4 presents the Energy Dispersive X-Ray Spectroscopy (EDS) results of the rubber provided by the manufacturer.

(5) Admixture: A polycarboxylate superplasticizer produced by Shanxi Feikese Materials Technology Co., Ltd. (Yuncheng, China) was used.
(6) Water: Standard laboratory tap water that meets the relevant specifications was used.

### 2.2. Mix Proportion of Materials

The water-to-binder ratio was designed as 0.43 in accordance with the Chinese industry standard *Test Code for Hydraulic Concrete* (SL/T 352-2020), which recommends a maximum water-to-binder ratio not exceeding 0.45 [23]. Following the determination of the water-to-binder ratio, and in accordance with the *Specification for Mix Design of Hydraulic Concrete* (DL/T 5330-2015), the sand-to-total aggregate ratio was set at 0.42 and the water-reducing admixture dosage at 0.6% (by mass of cementitious material) to maintain the fluidity of the concrete [24]. Rubber powder was incorporated at three volume fractions of the cementitious material: 0%, 5%, and 10%. Detailed mix proportion parameters are provided in Table 5.

### 2.3. Test Methods

#### 2.3.1. Compressive Performance Test

This study employed 100 mm × 100 mm × 100 mm cubic specimens, divided into three groups with three specimens each, to test the 28-day compressive strength of concrete with different rubber contents. After mixing according to the mix proportions and molding, the specimens were covered with plastic film and placed in a constant temperature and humidity curing room (20 ± 2 °C, RH ≥ 95%). They were demolded after 24 h and continued to undergo standard curing until 28 days.

(1) $$f_{cu}=\frac{F}{A}$$

where $f_{cu}$—compressive strength of concrete cubic specimens (MPa); F—failure load of specimens (N); A—bearing area of specimens (mm^2^).

#### 2.3.2. Anti-Impact Performance Test

Cylindrical specimens with a diameter of φ = 150 mm and a height of h = 64 mm were used for the impact tests. All mixing equipment was pre-wetted before mixing and cleaned promptly after use. Prior to formal mixing, a small amount of mortar was initially applied to the inner walls of the mixer and then scraped off to reduce paste adhesion losses. The batch volume was controlled between 1/4 and 3/4 of the mixer’s rated capacity. To promote uniform dispersion of the rubber powder, it was first pre-mixed with cement and water. Subsequently, coarse aggregate, fine aggregate, and cement were added sequentially. After starting the mixer, water was added gradually, with the total mixing time not exceeding 2 min. All performance testing and mold filling with vibration compaction were completed within 5 min after the end of mixing. After the specimens were compacted and shaped using a vibrating table, they were demolded and numbered after 24 h, then transferred to a standard curing room (temperature 20 ± 2 °C, relative humidity ≥ 95%) for curing periods of 3, 7, and 15 days.

Upon reaching the specified curing age, the specimens were retrieved from the curing location, wiped clean, and inspected to confirm the absence of visible damage before being mounted on the testing apparatus as required. A steel ball with a mass of 4.5 kg was freely dropped from a height of 457 mm onto the center area of the specimen. Each single impact was considered one cycle. The process was repeated until initial cracking appeared on the specimen surface, and the corresponding number of impacts was recorded as the initial crack resistance (N_1_). Impact testing continued until the specimen failed, and the total number of impacts was recorded as the failure impact number (N_2_).

(2) W=N⋅m⋅g⋅h

where *W*—Impact energy, J; *N*—Number of blows (number of failure impacts, N_2_); *h*—Drop height of the impact hammer, m; *m*—Mass of the impact hammer, kg; *g*—Gravitational acceleration (taken as 9.8), m/s^2^;

#### 2.3.3. Test Methods for Wear Rate and Abrasion Resistance Strength

The concrete abrasion test was conducted using the underwater steel ball method, with an adjustable stirring paddle speed set at 1200 r/min. A φ300 mm × 100 mm concrete specimen, cured under standard conditions for 28 days, was immersed in a constant-temperature water tank at 20 ± 2 °C for 48 h to achieve full saturation. After removal and surface drying, the saturated surface-dry (SSD) condition was determined according to the *Test Code for Hydraulic Concrete* (SL/T 352-2020) using the mass method: the mass M_0_ recorded when three consecutive weighings showed a variation of no more than 0.05% was taken as the baseline. Subsequently, the abrasion testing machine was operated at a speed of 1200 r/min for a continuous duration of 72 h to conduct the abrasion test. Based on the test results, the abrasion resistance strength and the wear rate were calculated.

The abrasion resistance of concrete was represented by the abrasion resistance strength, which was calculated according to Formula (3).

(3) $$f_{a}=\frac{TA}{∆M}$$

(4) $$∆M=(M_{0}-M_{f})$$

where $f_{a}$—Abrasion resistance strength of the concrete specimen, defined as the time required to wear away a unit mass per unit area, h/(kg/m^2^); T—Cumulative test duration, h; A—Area of the concrete specimen subjected to abrasion, m^2^; ∆M—Cumulative mass loss of the specimen after an abrasion duration of T, kg.

The wear rate of the concrete was calculated using Formula (5).

(5) $$L=\frac{M_{0}-M_{t}}{M_{0}}$$

where *L*—Wear rate, %; $M_{0}$—Mass of the specimen before testing, kg; $M_{t}$—Mass of the specimen after testing, kg.

#### 2.3.4. Three-Dimensional Topography Measurement

Abrasion action alters the surface morphology of concrete, resulting in a certain depth of wear on the abraded surface. To evaluate the wear depth and characterize the morphological changes, a simple three-dimensional topography scanning system utilizing a mechanical probe point measurement method can be employed to determine changes in the 3D topography of concrete [25,26,27]. The measurement principle is as follows: A three-dimensional Cartesian coordinate system is established with the center of the cylindrical concrete specimen as the origin. The *Z*-axis is perpendicular to the circular surface of the concrete, while the *X*-axis and *Y*-axis lie within the circular surface and are perpendicular to each other. A depth gauge, replacing a sensor, is used to measure the wear depth of the specimen. This depth gauge has a maximum measurement range of 120 mm along the *Z*-axis with an accuracy of 0.01 mm. By moving the depth gauge along the *X*-axis and the support bracket along the *Y*-axis, the wear morphology changes across the entire specimen surface can be measured. Considering both the accuracy of the wear morphology reflected by the number of measurement points and the required workload, a spacing of 5 mm between measurement points in both the X and Y directions is deemed most appropriate. Data measured at this spacing are used to plot the 3D topographic characteristic changes and calculate the wear depth of the concrete under different conditions [28,29].

#### 2.3.5. Microstructural Analysis

Modern microscopic analysis techniques, such as Scanning Electron Microscopy (SEM), were employed to systematically observe the morphology and distribution of hydration products in concrete after reaching the specified curing ages, in order to investigate the relationship between its microstructure and macroscopic properties.

## 3. Result and Discussion

### 3.1. Compressive Strength

**Figure 2:**
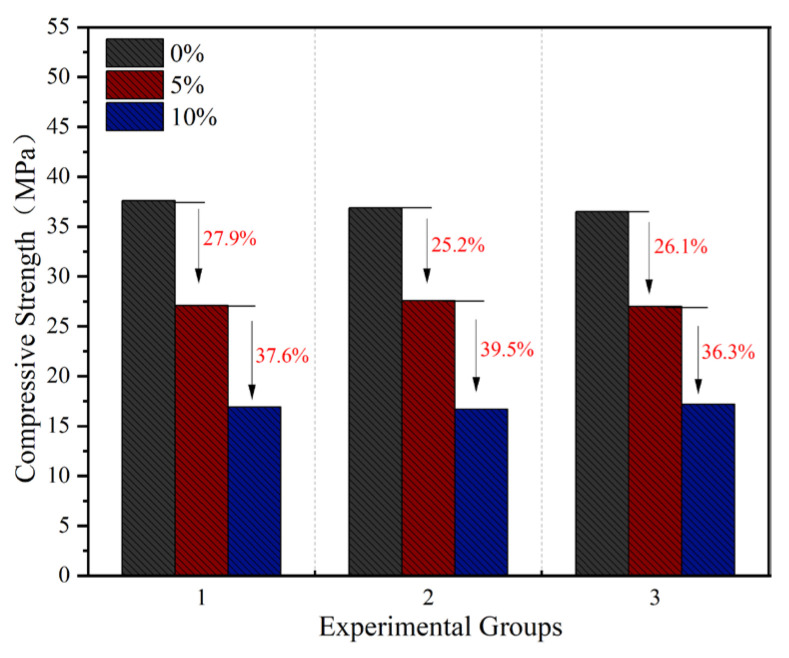
Compressive strength of concrete with different rubber powder contents.

As shown in Figure 2, the compressive strength of concrete exhibited a gradual decreasing trend with increasing rubber powder content. Specifically, when the rubber powder content was 5%, the compressive strength decreased by approximately 26.4% on average compared to the reference group (without rubber powder). When comparing the 10% content group to the 5% content group, the compressive strength showed an average decrease of about 37.8%. The primary reasons for this strength reduction may include the following aspects [30,31]:

(1) The elastic modulus of rubber particles is significantly lower than that of the surrounding cement matrix. Under external load, the rubber particles undergo larger elastic deformation, leading to stress concentration phenomena, thereby reducing the overall stiffness of the material.
(2) The hydrophobic surface of rubber exhibits poor compatibility with the hydrophilic cement paste, resulting in a weak interfacial transition zone (ITZ). This zone is prone to become the path for micro-crack initiation and propagation.
(3) The incorporation of rubber powder may increase the air content and reduce the compactness of the concrete, raising the overall porosity. This exacerbates stress concentration effects and promotes crack propagation.

However, crumb rubber reduces concrete’s compressive strength, requiring optimized content to balance strength with recycling benefits.

### 3.2. Impact Resistance Test

**Figure 3:**
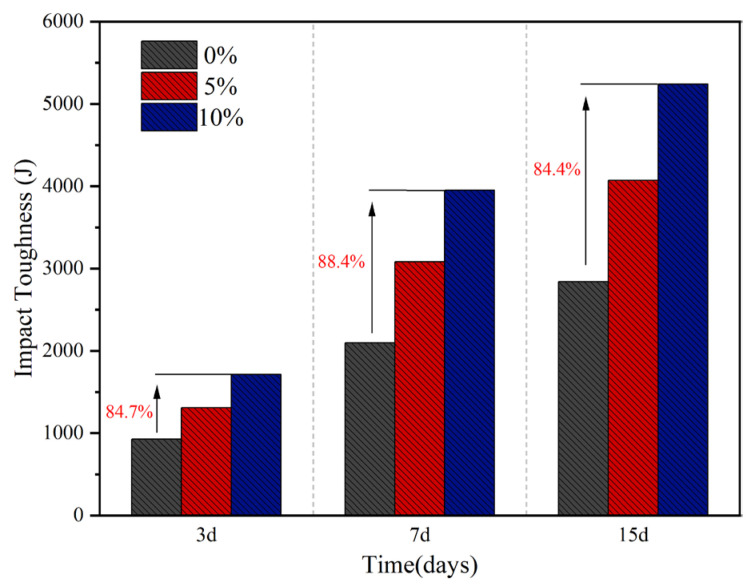
Impact toughness of concrete with different rubber powder contents.

As shown in Figure 3, the experimental results indicate that the incorporation of rubber powder significantly enhances the impact toughness of concrete, and this property further improves with increasing rubber powder content. When the rubber powder content was 10%, compared to the control group without rubber powder, the impact toughness of the concrete specimens increased by 84.7%, 88.4%, and 84.4% after 3, 7, and 15 days of curing, respectively. These results demonstrate that the addition of rubber powder effectively improves the impact resistance of cement concrete. The primary reasons for this enhancement may include the following aspects [32]:

(1) The low elastic modulus of rubber allows for large deformation under impact loading, thereby absorbing and dissipating energy and effectively reducing the peak stress.
(2) As a soft phase within the matrix, rubber particles act as obstacles, causing crack deflection or branching during propagation, which delays macroscopic failure. Additionally, as cracks pass through the cement-rubber interface, they must overcome the interfacial bond strength and the deformation of the rubber itself, further consuming impact energy.
(3) The rubber particles and cement matrix form a composite system that enhances overall toughness through coordinated deformation. Furthermore, the micro-porous structure resulting from the incorporation of rubber helps to absorb energy and alleviate local stress concentration.

### 3.3. Damage Layer Thickness

**Figure 4:**
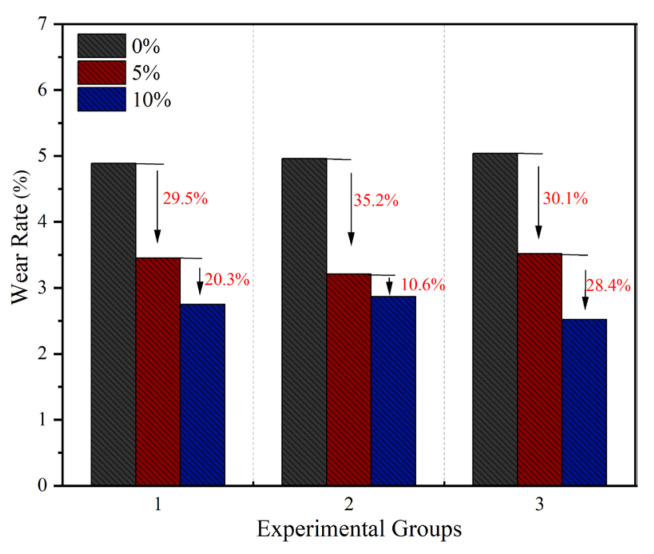
Wear rate of concrete with different rubber powder contents.

As shown in Figure 4, the experimental results demonstrate that the average wear rate of all concrete specimens incorporating waste rubber powder was lower than that of the reference group without rubber powder (average wear rate: 4.89%). When the rubber powder content was 5%, the average wear rate of the specimens was approximately 3.39%, representing a significant reduction of about 30.7% compared to the reference group. This indicates that even at a low content, rubber particles can effectively enhance the abrasion resistance of concrete. When the content was increased to 10%, the average wear rate further decreased to 2.81%; however, the reduction rate narrowed to 17.1% compared to the specimens with 5% content, suggesting that the rate of performance improvement slows down with increasing content.

The above phenomenon can be attributed to microstructural evolution induced by changes in rubber powder content. At lower contents (e.g., 5%), rubber particles can be relatively uniformly dispersed within the cement matrix, absorbing impact energy through elastic deformation, improving pore structure, and enhancing the bond between aggregates, thereby significantly reducing the wear rate. However, as the content increases (e.g., 10%), rubber particles are prone to agglomeration, with enhanced interparticle interactions leading to weakened interfacial bonding with the cement matrix, reduced filling of active pores by the paste, and exacerbated stress concentration. Consequently, the magnitude of further performance improvement is diminished.

In summary, a distinct two-stage relationship exists between rubber powder content and the abrasion resistance of concrete: the initial stage shows significant performance improvement, but when the content exceeds a certain threshold, agglomeration effects and increased interfacial defects cause the efficiency of performance enhancement to decline.

**Figure 5:**
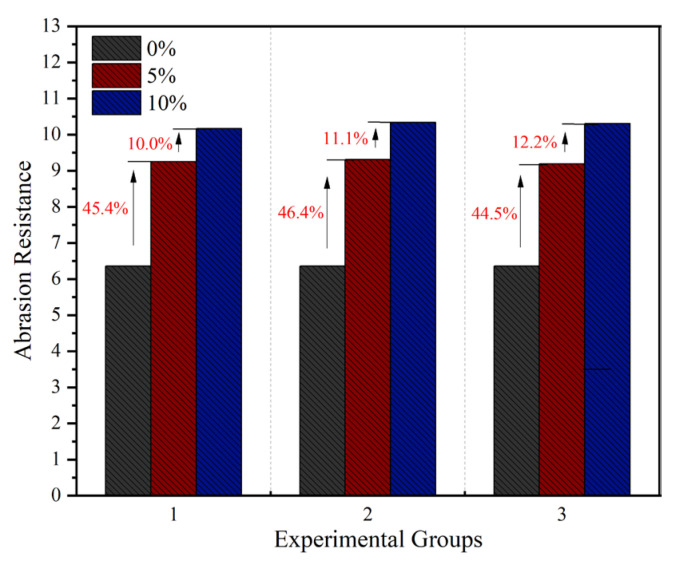
Abrasion resistance strength of concrete with different rubber powder contents.

As shown in Figure 5, the experimental results demonstrate that incorporating waste rubber powder significantly improves the abrasion resistance strength of concrete. The average abrasion resistance strength of the reference concrete without rubber powder was 6.36 h/(kg/m^2^), while all specimens containing rubber powder exhibited better performance than the control group.

Specifically, when the rubber powder content was 5%, the abrasion resistance strengths of the three specimen groups were 9.25, 9.31, and 9.19 h/(kg·m^2^), respectively, with an average value of 9.25 h/(kg·m^2^). This represents an increase of approximately 45% compared to the reference group. When the content was increased to 10%, the corresponding strength values were 10.17, 10.34, and 10.21 h/(kg·m^2^), respectively, averaging 10.24 h/(kg·m^2^). Although this shows a further improvement over the 5% content group, the rate of increase narrowed to about 11%. These results indicate that the rate of improvement in abrasion resistance strength significantly slows as the rubber powder content increases from 5% to 10%.

This phenomenon is closely related to the dispersion state of the rubber powder in the cement matrix and the interfacial effects. At a lower content (5%), the rubber powder particles are uniformly distributed. Their elastic behavior helps convert impact kinetic energy into elastic potential energy, delaying crack initiation. Meanwhile, the filling effect of the particles enhances the material’s compactness. However, at a higher content (10%), the rubber powder tends to agglomerate, causing stress concentration and interfacial weakening. Excessive rubber particles disrupt the continuity of the cement matrix, weaken the bond between aggregates, and make the “replacing rigidity with flexibility” effect more pronounced, thereby limiting further improvement in abrasion resistance [33].

### 3.4. The Hydration Products

**Figure 6:**
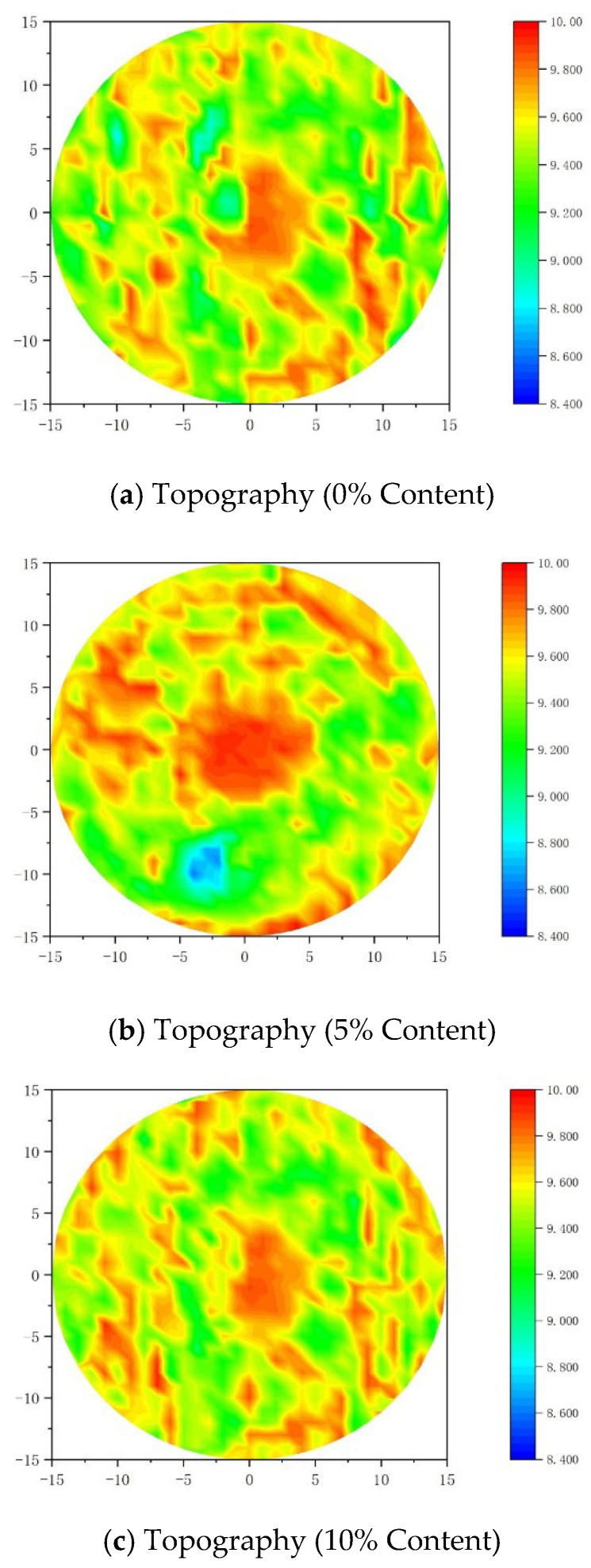
Three-dimensional topography of concrete surfaces with different rubber powder contents.

As shown in Figure 6, a set of specimens from the experimental group was selected for three-dimensional topography measurement. In the control group specimen, the surface morphology in the central area remained relatively intact, with an abrasion depth of less than 4 mm, corresponding to the red areas in the figure. Abrasion severity gradually increased from the center towards the periphery. The edge areas showed a relatively irregular distribution, with surface abrasion depths mostly between 8–10 mm, corresponding to the green and light blue areas in the figure. These observations indicate that abrasion damage initiated at the specimen edges and progressively extended towards the central region.

The concrete specimen with 5% rubber powder content experienced reduced abrasion damage. Its morphological characteristics in the figure show a relatively larger undamaged central area, with the intact region (represented in red) significantly larger than that of the control group. The bond between mortar and aggregate in the specimen was noticeably better than in the reference group, and the surface abrasion depth was primarily concentrated in the 4–8 mm range. However, the local maximum abrasion depth at the specimen edges exceeded 14 mm, significantly higher than that of the reference concrete. This is likely because, at this low content, the rubber powder’s improvement effect on the concrete’s overall toughness was not fully realized, and the enhancement was limited, leading to relatively severe erosion in localized areas under high-speed abrasion.

Compared to the control group, the concrete specimen with 10% rubber powder content exhibited a larger central intact area, a more extensive transition zone, and abrasion depths mainly concentrated in the 4–6 mm range, with a more uniform overall distribution than the 5% group. These results indicate that the uniform dispersion of rubber particles within the concrete effectively enhanced the material’s overall toughness and promoted a more uniform distribution of impact stress over a larger area. The mechanism can be attributed to the synergistic deformation behavior between the rubber powder and the cement mortar matrix: through a viscoelastic response, locally concentrated erosion energy is transformed into distributed dissipated energy, thereby inhibiting the formation and propagation of macroscopic spalling pits [34].

### 3.5. Microscopic Analysis and Discussion

**Figure 7:**
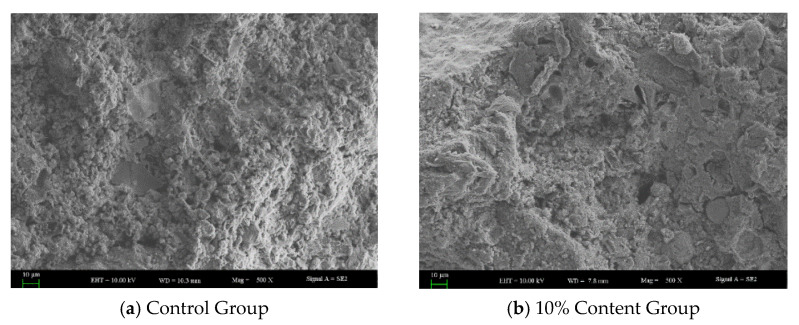
Pore structure of concrete.

**Figure 8:**
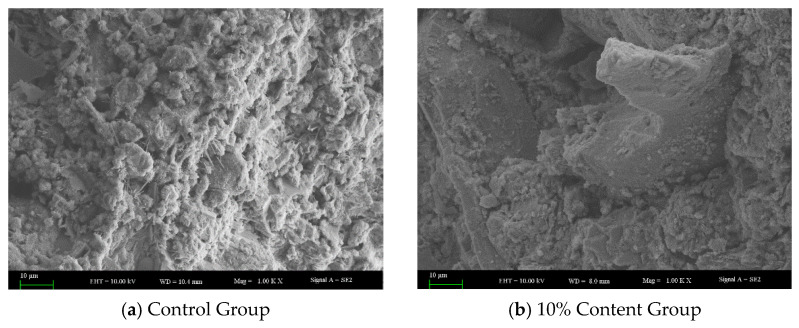
C-S-H distribution in concrete.

As shown in Figure 7 and Figure 8, The reference concrete without rubber powder exhibits a relatively ideal microstructure, characterized by a uniform pore distribution, small pore sizes, and low total porosity, along with a dense and continuously distributed C-S-H gel structure, which collectively contribute to its satisfactory mechanical properties. However, the incorporation of rubber powder significantly alters the microstructure of the concrete. On one hand, as an inert component, rubber powder replaces part of the space that would otherwise be occupied by hydration products, resulting in a dilution effect that directly reduces the amount of C-S-H gel per unit volume. On the other hand, rubber particles hinder the migration of water towards unhydrated cement particles, thereby inhibiting further cement hydration. These factors collectively lead to a reduction in the formation of C-S-H gel, a decrease in its distribution continuity, and an increase in total porosity, particularly in the form of a greater volume of large-sized pores. The deterioration of the microstructure—specifically, the reduction in C-S-H gel and the coarsening of the pore structure—is identified as the primary reason for the significant decline in the compressive strength of rubberized concrete.

**Figure 9:**
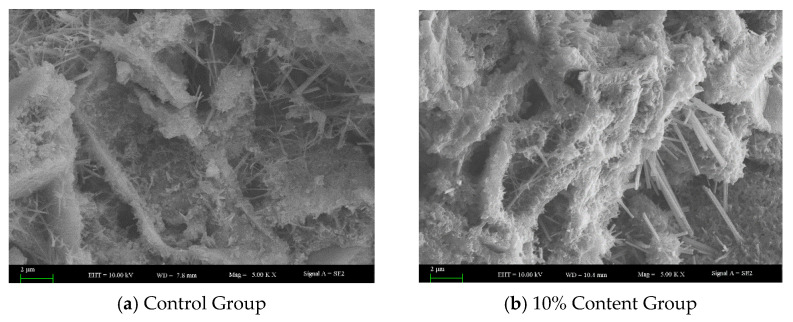
Comparison of ettringite formation.

As shown in Figure 9, compared to the control group, the 10% content group exhibited less ettringite formation. Ettringite contributes to matrix densification by filling pores during the early hydration stage of cement. However, if delayed ettringite formation occurs after the concrete has hardened, the crystal growth can generate internal stress, leading to reduced material toughness, diminished abrasion resistance, and the initiation of microcracks. The incorporation of rubber powder introduces inert rubber particles that, at the microscale, partially hinder the dispersion and hydration of cement particles, restrict the nucleation and growth space of ettringite crystals, and potentially retard the migration rate of relevant ions, thereby modulating the hydration kinetics. This mechanism suppresses the formation of delayed ettringite to some extent, consequently contributing to the enhancement of the concrete’s toughness and impact-abrasion resistance.

## 4. Conclusions

This study investigated the effects of waste rubber powder on the mechanical properties and abrasion resistance of concrete. Based on the experimental results, the following conclusions can be drawn:

(1) The addition of rubber powder reduces the compressive strength of concrete through synergistic physical effects. Microscopically, rubber acts as an inert filler, diluting C–S–H gel formation and inhibiting cement hydration, which increases porosity—especially macropores—and weakens microstructural continuity. Macroscopically, the low stiffness of rubber causes stress concentration, while its hydrophobic nature creates a weak interfacial transition zone (ITZ) that promotes cracking. These microstructural and interfacial defects, compounded by increased air content, collectively account for the strength reduction.
(2) Rubber particles improve concrete impact resistance through three mechanisms: energy absorption via elastic deformation, crack deflection through interface debonding, and stress relief from induced micro-porosity, collectively enhancing toughness and energy dissipation. The incorporation of rubber powder exerts a physical inhibitory effect on the cement hydration process, restricting the adequate dispersion and hydration reaction of cement particles, thereby significantly reducing the formation of delayed ettringite. This effect effectively mitigates the crystallization-induced internal stress generated by the formation of delayed ettringite in the later hardening stages, consequently alleviating the resulting deterioration in material toughness. As a result, the abrasion and impact resistance of rubberized concrete are significantly enhanced.
(3) Analysis of the wear rate, abrasion resistance strength, and three-dimensional topography revealed a significant improvement in the abrasion resistance of rubber powder concrete. This enhancement is attributed to the reduction in ettringite formation due to the incorporation of rubber powder, which decreased late-stage cracking. Furthermore, the uniform dispersion of rubber powder within the concrete matrix considerably enhanced the overall toughness of the material and promoted a more uniform distribution of impact stress over a wider area. The underlying strengthening mechanism primarily stems from the synergistic deformation behavior between the rubber particles and the cement mortar matrix: the rubber phase, through its viscoelastic response, transforms locally concentrated erosion energy into distributed dissipated energy, thereby effectively suppressing the initiation and propagation of macroscopic spalling pits.

## Figures and Tables

**Table 1:** Main Physical Properties of Cement.

Density (g/cm^2^)	Blaine Specific Surface Area (m^2^/kg)	Water Requirement for Standard Consistency (%)	Setting Time (min)		Soundness	Flexural Strength (MPa)		Compressive Strength (MPa)	
			Initial Setting Time	Final Setting Time		3d	28d	3d	28d
3.1	364	26.9	182	224	Qualified	5.1	8.6	22.1	51.0

**Table 2:** Physical Properties of Sand.

Apparent Density (g/cm^3^)	Water Absorption (%)	Fineness Modulus	Clay Content (%)	Sulfide and Sulfate Content (%)	Organic Matter Content
2.65	1.2	2.9	1.2	0.3	Lighter than the standard color

**Table 3:** Physical Properties of Coarse Aggregate.

Apparent Density (g/cm^3^)	Water Absorption (%)	Crushing Value (%)	Sulfide and Sulfate Content (%)	Organic Matter Content
2.68	0.8	3	8	Lighter than the standard color

**Table 4:** EDS Analysis Results of Waste Rubber Powder.

Element	C	O	Ca	Zn	S	Na	Cl	K	Si	Fe	Al
Content (%)	77.69	18.71	1.1	0.39	0.56	0.51	0.28	0.25	0.3	0.12	0.09

**Table 5:** Mix Proportion Design of Concrete.

Water-To-Binder Ratio	Water (kg/m^3^)	Cement (kg/m^3^)	Sand Ratio	Fine Aggregate (kg/m^3^)	Crushed Stone (kg/m^3^)	Superplasticizer (%)	Waste Rubber Powder (%)
0.43	168.40	387.50	0.42	801.00	1109.40	0.6	0.0
0.43	159.98	368.13	0.42	760.95	1053.93	0.6	5.0
0.43	151.56	348.75	0.42	720.90	998.46	0.6	10.0

## Data Availability

The original contributions presented in the study are included in the article, further inquiries can be directed to the corresponding author.
